# Bleomycin-Treated Chimeric Thy1-Deficient Mice with Thy1-Deficient Myofibroblasts and Thy-Positive Lymphocytes Resolve Inflammation without Affecting the Fibrotic Response

**DOI:** 10.1155/2015/942179

**Published:** 2015-08-02

**Authors:** Pazit Y. Cohen, Raphael Breuer, Philip Zisman, Shulamit B. Wallach-Dayan

**Affiliations:** ^1^Lung Cellular and Molecular Biology Laboratory, Institute of Pulmonary Medicine, Hadassah-Hebrew University Medical Center, Jerusalem 91120, Israel; ^2^Department of Pathology, Boston University School of Medicine, Boston, MA 02118, USA

## Abstract

Lung fibrosis is characterized by abnormal accumulation of fibroblasts in the interstitium of the alveolar space. Two populations of myofibroblasts, distinguished by Thy1 expression, are detected in human and murine lungs. Accumulation of Thy1-negative (Thy1^−^) myofibroblasts was shown in the lungs of humans with idiopathic pulmonary fibrosis (IPF) and of bleomycin-treated mice. We aimed to identify genetic changes in lung myofibroblasts following Thy1 crosslinking and assess the impact of specific lung myofibroblast Thy1-deficiency, in vivo, in bleomycin-injured mouse lungs. Thy1 increased in mouse lung lymphocytes following bleomycin injury but decreased in myofibroblasts when fibrosis was at the highest point (14 days), as assessed by immunohistochemistry. Using gene chip analysis, we detected that myofibroblast Thy1 crosslinking mediates downregulation of genes promoting cell proliferation, survival, and differentiation, and reduces production of extracellular matrix (ECM) components, while concurrently mediating the upregulation of genes known to foster inflammation and immunological functions. Chimeric Thy1-deficient mice with Thy1^+^ lymphocytes and Thy1^−^ myofibroblasts showed fibrosis similar to wild-type mice and an increased number of CD4/CD25 regulatory T cells, with a concomitant decrease in inflammation. Lung myofibroblasts downregulate Thy1 expression to increase their proliferation but to diminish the in vivo inflammatory milieu. Inflammation is not essential for evolution of fibrosis as was previously stated.

## 1. Background

Idiopathic pulmonary fibrosis (IPF) is a nonneoplastic pulmonary disease characterized by the formation of scar tissue within the lungs, in the absence of any known provocation [1, 2]. Fibroblasts are central to both wound healing and the pathogenesis of organ tissue fibrosis. Although it remains unclear whether fibroblast proliferation is the primary mechanism of pathogenesis in IPF or whether it is a reactive process to another form of lung tissue damage, selective deletion of fibroblasts is sufficient to prevent fibrosis after injury [3]. The intratracheal instillation (IT) of bleomycin into mice is used as an in vivo experimental model to study underlying mechanisms of lung fibrosis and inflammation [4].

Lung fibroblasts can be divided into subpopulations on the basis of size and shape, cytokine profiles, lipid content, and cell surface protein expression [5, 6]. The most extensively characterized in vitro model of fibroblast heterogeneity is based on surface expression of Thy1 [5, 7]. It has been shown in mice and humans that Thy1-positive (Thy1^+^) and Thy1-negative (Thy1^−^) fibroblasts differ with respect to cytokine [8–12] and growth factor responses [13, 14], as well as cell migration patterns [15].

Thy1 is a 25–37 kD glycosylphosphatidylinositol- (GPI-) anchored cell surface protein that belongs to the immunoglobulin-like gene super family [16]. Thy1 is expressed and distributed differently among species and among tissues of the same species; however, it is present on brain cells and fibroblasts of all species studied thus far [17]. In mice, Thy1 is also found on other cell types, including thymocytes and peripheral T cells [17, 18]. The wide distribution of Thy1 suggests that it has distinct functions in different tissues and species. Immune cells such as T lymphocytes were previously reported to be involved in both the attenuation and the promotion of fibrosis. These contradictory observations are most likely a reflection of the phenotypic heterogeneity of involved T cells, as reviewed by Re et al. [19] and Luzina et al. [20].

In this study, we aimed to define the molecules and pathways involved in inflammation and fibrosis that are affected by Thy1 crosslinking on lung myofibroblasts in vitro and specifically by its deficiency in lung myofibroblasts in vivo, in the experimental model of bleomycin-induced fibrosis in mouse lungs, excluding the role of Thy1 on lymphocytes.

## 2. Materials and Methods

### 2.1. Animals

Male, 11 to 12 weeks old, C57BL/6J mice (Jackson Laboratory, Bar Harbor, ME) and C57BL/6J Thy1-deficient mice (kindly provided by Professor R. J. Morris, Laboratory of Neurobiology, National Institute for Medical Research, London, UK) were used.

At 12–14 weeks, body weight for the two types of mice was similar. Histological sections of lung, heart, brain, colon, liver, and kidney were studied. No differences were noted in histological sections or in lung hydroxyproline contents for the two types. Bronchoalveolar cellularity was also similar in terms of the number of cells per mL, with 99% macrophages, and 1% lymphocytes or neutrophils.

All animal procedures were approved by the Hebrew University-Hadassah Medical School Animal Care Committee. Mice were housed in a specific pathogen-free environment.

#### 2.1.1. Chimeric Mice and Lymphoid Organ Cell Transplantation

We created a C57BL/6J Thy1-deficient chimeric mouse containing wild-type (WT) (Thy1^+^) lymphoid organ cells. Lymphoid tissues in the Thy1-deficient mice were ablated, and the mice were reconstituted with hematopoietic cells isolated from WT mouse lymphoid organs as we previously performed and detailed [21]. WT mice reconstituted with hematopoietic cells isolated from lymphoid organs of WT mice served as control group. One day prior to cell transplantation, 6-week-old C57BL/6 mice were subjected to total body irradiation (750 cGy, dose rate of 179 cGy/min) delivered by a linear accelerator (Clinac G6, Varian Medical Systems, Palo Alto, CA, USA) with a source-to-skin distance of 80 cm. One day after irradiation, mice received syngeneic lymphoid organ cells (spleen and lymph nodes, 50–100 × 10^6^) obtained from WT- C57BL/6-based mice. Following adoptive transfer (30 days) and engraftment confirmation by FACS analysis (Figure 3(a)), chimeric mice were subjected to intratracheal instillation (IT) of bleomycin.

### 2.2. Intratracheal Instillation

Mice were anesthetized by intraperitoneal (IP) injection of 0.05–0.07 mL of 40 mg/mL ketamine (Ketalar, Parke-Davis, Pontypool, Gwent, UK) and 0.5 mg/mL droperidol (Inapsine, Janssen Pharmaceutica, Beerse, Belgium). The skin and subcutaneous tissues overlying the proximal portion of the trachea were exposed by a 5 mm transverse incision to allow for direct external visualization of the trachea. A metal cannula fitted to a tuberculin syringe was carefully passed into the trachea. A dose of 0.06–0.08 units of bleomycin (H. Lundbeck, Copenhagen, Denmark) dissolved in 0.1 mL of saline solution, or 0.1 mL of saline alone, was slowly injected.

### 2.3. Quantitative Assessment of Fibrotic Lung Injury

Mice were killed with a lethal dose of pentobarbital at 1, 3, 7, or 14 days after IT bleomycin. The abdominal aorta was transected, and the animal was exsanguinated. To eliminate blood, lungs were perfused with normal saline through the right ventricle and bronchoalveolar lavage (BAL) was performed. A polyethylene cannula (PE 205; Clay Adams, Parsippany, NJ, USA) was placed in the trachea, and 3 mL of normal saline was slowly injected and withdrawn.

Lung injury was assessed quantitatively, as we have previously described [22] by bronchoalveolar lavage (BAL) cellularity, semiquantitative morphological index (SMI) studies of lung fibrosis, and quantitative lung collagen measurements.

### 2.4. Lung Collagen

Lung collagen was assessed in the right lung using the Sircol Collagen Assay kit (Biocolor, Belfast, Northern Ireland), as described previously [23, 24]. This method measures newly synthesized collagen that has not been extensively cross-linked. Briefly, the upper lobe of the right lung was homogenized in 5 mL of 0.5 molar acetic acid containing 1 mg pepsin (Sigma Aldrich, St Louis, MO, USA) per 10 mg tissue residue. Each sample was incubated at room temperature for 24 h, with stirring. After centrifugation, 100 *μ*L of each supernatant was assayed, 1 mL of Sircol dye reagent that specifically binds to collagen was added to each sample, and the sample was mixed for 30 min. After centrifugation, the pellet was suspended in 1 mL of the alkali reagent (0.5 molar NaOH) included in the kit, and optical density was evaluated at 540 nm with a spectrophotometer. Values in the test samples were compared to values obtained with collagen standard solutions provided by the manufacturer, which were used to construct a standard curve. Collagen results were expressed in micrograms.

### 2.5. Lung Cell Isolation and Myofibroblast Culture

Lungs were removed, minced, and incubated (37°C, 5% CO_2_ air) for 45 min in PBS containing 1 mg/mL collagenase (C0130, Sigma Aldrich). After enzyme treatment, lung tissue was gently passed through a cell dissociation sieve (Sigma Aldrich) or 40 *μ*m nylon mesh filters (Falcon, Becton Dickinson, Franklin Lakes, NJ, USA) and then washed twice in PBS. For myofibroblast culture experiments, lung cells were resuspended in fibroblast culture medium. Cell cultures were incubated at 37°C in 5% humidified CO_2_. Typically, within 1 week of culture initiation, more than 95% of the cells are morphologically myofibroblasts.

Cells were passaged every 5 days by dissociating monolayers with a mild trypsin solution (Biological Industries, Beit Haemek, Israel). After initial cultures were established, fibroblasts obtained on passages 2 through 6 were used.

### 2.6. Immunostaining of Frozen Tissue Sections

The left lung was removed and frozen in Tissue-Tek O.T.C. compound (Sakuar Finetek, Zoeterwoude, Netherlands). Frozen tissue blocks were cut to provide 4–6 *μ*m sections, which were fixed in acetone for 5 min at −20°C and air dried. Endogenous peroxidase activity was blocked with immersion of sections in 0.3% H_2_O_2_, 0.1% NaN_3_ in methanol for 20 min at room temperature (RT) and washed three times. Nonspecific reactions were blocked with 10% FCS for 10 min at RT. After washes with PBS, sections were incubated with rat anti-Thy1 (catalog number 550543, BD Biosciences/Pharmingen, San Jose, CA, USA) for 2 h at RT. After PBS washing, sections were reincubated with universal immunoperoxidase polymer for mouse tissue sections in anti-rat primary antibody (Histofine, Nichirei Biosciences, Tokyo, Japan) for 30 min at RT and again reincubated for a 10 min in peroxidase substrate (Dako Kit, Dako, Glostrup, Denmark). The sections were counterstained with hematoxylin (Zymed Kit, Zymed Laboratories, San Francisco, CA) and mounting was added to the slides (Zymed Kit).

### 2.7. Immunostaining by Flow Cytometry

Cells were harvested by rubber policeman. Myofibroblasts (0.5 × 10^6^) were incubated in FACS buffer (3% FCS in PBS), with 1 *μ*g/100 *μ*L PE-conjugated anti-Thy1 (CD90) mAb (BD Biosciences/Pharmingen) for 30 min at RT. The cells were then washed with FACS buffer and analyzed by flow cytometry.

### 2.8. Myofibroblast Thy1 Crosslinking

Subconfluent myofibroblasts were stimulated with anti-Thy1 G7, which has previously been shown to activate T cells [25] or with anti-rat IgG2C*κ* isotype control (BD Biosciences/Pharmingen). Both stimulants were added to myofibroblasts at concentrations ranging from 1 to 20 *μ*g/mL, together with recombinant protein G crosslinker (Sigma Aldrich) at the same concentration.

### 2.9. Immunofluorescence Staining of CD4, CD8, and CD25 in Lung Sections

Paraffin lung sections were stained as we have previously described in detail [24]. Briefly, deparaffinized lung sections were incubated overnight at 4°C with APC-conjugated CD4/FITC-conjugated CD25 or FITC-conjugated CD8 (BD Biosciences/Pharmingen), diluted at 1 : 200 in 1% BSA PBS Tween 0.05%. After washing, the slides were analyzed by confocal microscopy (Axio Scope 2; Carl Zeiss AG, Oberkochen, Germany). The fluorescence ratio was analyzed using the Ariol system [26]. The relative staining ratio was calculated by dividing the number of stained cells by the unstained cells in a certain analyzed area.

### 2.10. Immunohistochemical *α*SMA Staining of Lung Tissue Sections

Paraffin sections were stained as we have previously described in detail [24]. Briefly, deparaffinized lung sections were incubated overnight at 4°C with polyclonal mouse anti-SMA (DAKO) diluted 1 : 200 in 1% BSA PBS Tween 0.05%. The Envision Detection System (DAKO) containing secondary anti-mouse horseradish peroxidase-conjugated antibody and 3,3′-diaminobenzidine as a substrate was used for staining detection. The quantitative analysis of DAB staining was analyzed using the Ariol system. The relative staining ratio was calculated by dividing the number of stained cells by the unstained cells in a certain analyzed area.

### 2.11. Sirius Red Staining for Collagen (Type I and IV)

Deparaffinized lung sections were stained using Picro Sirius Red Stain Kit (Abcam, Cambridge, UK) as was previously described [27]. Red sirius staining was analyzed using the Ariol system. Comparisons were made between the two groups of IT bleomycin. The relative staining ratio was calculated by dividing the number of stained cells by unstained cells in a certain analyzed area.

### 2.12. RNA Isolation

Total cellular RNA was isolated from myofibroblasts in culture using TriReagent (catalog number T9424, Sigma Aldrich), according to the protocol supplied by the manufacturer. To assess RNA integrity and exclude DNA contamination, an aliquot of each sample was analyzed by electrophoresis on a 1% agarose gel stained with ethidium bromide. Purity and quantitation of RNA were assessed by spectrophotometer.

### 2.13. RT-PCR

RNA was reverse transcribed to cDNA using an avian myeloblastosis virus-RT base protocol and random primers, as well as poly dT (Reverse Transcription System; Promega, Madison, WI, USA). One microgram of each sample was uniformly used for reverse transcription. cDNA was diluted in a final volume of 200 *μ*L with nuclease-free water.

### 2.14. Microarray

Total RNA was extracted and used as a template for double-stranded cDNA synthesis as previously described [28].

### 2.15. Preparation of Labeled cDNA

Labeling was performed using the Low RNA Input Linear Amplification Kit PLUS (One-Color, Agilent, Santa Clara, CA, USA). Briefly, Cy3-labeled double-stranded cDNA synthesis was performed using with an oligo(dT)24 primer containing a T7 RNA polymerase promoter site added to the 3′ and cyanine 3-CTP. cDNA was used as a template for generation of cRNA for hybridization.

### 2.16. Hybridization of Microarrays

After purification and fragmentation, aliquots of each sample were hybridized to Whole Mouse Genome 4 × 44 K Multipack Arrays (Agilent) with probes for 41,000 unique transcripts. In our experience, these arrays have demonstrated superior reproducibility and were recently shown to have superior sensitivity [29]. After hybridization, each array was sequentially washed and scanned (DNA Microarray Scanner, Agilent). Individual arrays were visually inspected for hybridization defects using manufacturer recommended quality control procedures and read out with Agilent Feature Extraction Software. Bioconductor (Bioconductor Project, Seattle, WA, USA) was used to normalize the processed signal [30]. Probes with annotations for entrez gene ID were extracted, and cyclic LOESS was applied to normalize gene expression signals. In cases of redundant probes, we took the average over values representing gene expression levels.

### 2.17. Gene Chip Analysis

Differentially expressed genes were identified by volcano analysis, using a threshold of >2-fold changes in expression. *P* < 0.05 was considered statistically significant. The analysis of biological processes affected by Thy1 in lung, myofibroblasts was performed using Onto-Express software (Open Channel Foundation, Chicago, IL, USA).

### 2.18. Statistical Analysis

The Mann-Whitney test was performed for comparisons of nonparametric data. When multiple pair-wise comparisons involving two groups were performed, the Bonferroni correction of the *P* value was used. The nonparametric Kruskal-Wallis test was performed for comparing more than two groups. *P* ≤ 0.05 or less was considered statistically significant.

## 3. Results

### 3.1. Thy1 Expression in Lung Tissue Is Increased at Times of Active Fibrosis following Bleomycin IT but Specifically Decreased in Lung Myofibroblasts

**Figure 1:**
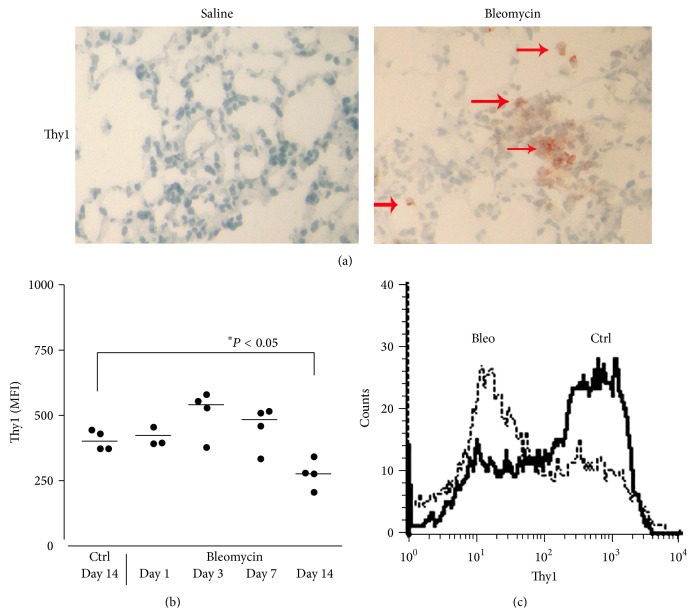
Thy1-positive total lung cells are increased during experimental fibrosis; however, the proportion of myofibroblasts that are Thy1-positive is decreased. (A) Immunohistochemistry of Thy1 staining (arrows), in the lungs of saline- vs. bleomycin-treated mice, 14 days following instillation. Representative results. (B) Lung myofibroblasts were isolated from control saline-treated (Ctrl) and bleomycin-treated mice at 1, 3, 7 and 14 days following IT. A graphical presentation of Thy1 expression FACS analysis was performed using PE-conjugated anti-Thy1 (CD90) mAb. The differences between control (Ctrl) saline-treated and bleomycin-treated mouse lung myofibroblasts on day14 were significant. ^*∗*^ *P* < 0.05, *n* = 4. (C) Histogram plot showing a representative result of assessment of Thy1 expression in myofibroblasts from control (Ctrl) saline-treated mice compared to Thy1 expression in myofibroblasts from bleomycin-treated mice at day 14 following IT. Results were similar in 2 different experiments (*n* = 4).

Thy1 expression was assessed by immunohistochemistry in frozen lung tissue sections of saline- and bleomycin-treated mice 14 days following instillation, the point of peak fibrosis [4]. When compared to saline-treated mice, Thy1 expression in the total lung was increased following bleomycin instillation (Figure 1(a)).

We sought to specifically determine Thy1 expression in lung myofibroblasts at different stages following bleomycin injury (1, 3, 7, and 14 days following IT bleomycin). Flow cytometry analysis using PE-conjugated anti-Thy1 mAb demonstrated that Thy1 expression in lung myofibroblasts is significantly decreased by day 14 (Figures 1(b) and 1(c), segregated histograms), indicating an increase in the proportion of Thy1^−^ myofibroblasts in the total lung myofibroblast population at later stages following bleomycin injury, when fibrosis develops [4].

### 3.2. Genomic Analysis Shows an Increased Expression of Proinflammatory Genes in Lung Myofibroblasts following Direct Thy1 Crosslinking-Induced Activation

**Figure 2:**
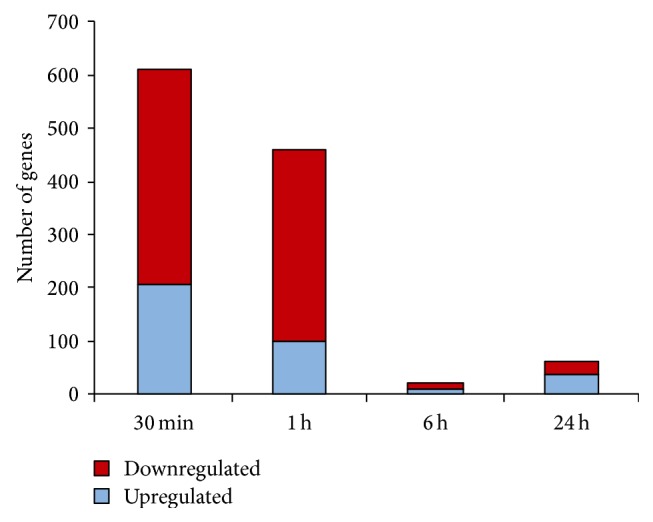
The number of genes in lung myofibroblasts with significant changes in expression at varying time points following Thy1 stimulation. Graphic presentation of GeneChip microarray results obtained from primary myofibroblast RNA extracts following stimulation with G7 anti-Thy mAb (5 *μ*g/mL) or control IgG isotype for 30 min, 1 h, 6 h, or 24 h. The microarray data were processed by volcano analysis. Upregulated genes (blue), downregulated genes (red).

To assess the effects of Thy1 activation on lung myofibroblasts, we analyzed gene expression patterns using GeneChip microarrays (Affymetrix, Santa Clara, CA, USA) in samples of RNA extracted from lung primary lung myofibroblasts at different time points before and after Thy1 crosslinking by G7 anti-Thy1 mAb 10 *μ*g/mL G7, compared to crosslinking with control matched-IgG isotype, for 30 min and 1, 6, and 24 hours. Genes with significant expression changes following Thy1 crosslinking-induced activation were detected following volcano analysis, using a threshold of >2-fold changes of gene expression over the untreated control and *P* < 0.05 as the test for significance. Most changes occurred very quickly, from 30 min-to-1 h following Thy1 stimulation. The number of downregulated genes was larger than the number that was upregulated (Figure 2).

An analysis of genes related to different biological processes affected by Thy1 in lung myofibroblasts was performed using Onto-Express software. Pathways “downregulated” by Thy1 crosslinking are shown in Table 1; “upregulated” pathways are found in Table 2.

In Table 3, we present selectively grouped genes according to pathways that were shown to influence myofibroblast proliferation and extracellular matrix (ECM) production/interaction and differentiation. In addition, changes in myofibroblast inflammatory and immunological genes were grouped. Note that a single gene may participate in more than one pathway.

While most of the genes involved in proliferation, ECM production and interaction, and differentiation were decreased, genes associated with inflammation and immunological functions were increased, following Thy1 stimulation. We have previously showed FasL gene overexpression on lung myofibroblasts following Thy1 crosslinking, verifying GeneChip results at the RNA level by qPCR and at the protein level by Western blot analyses [28].

### 3.3. Decreased Inflammation with Similar Fibrosis Development following IT Bleomycin in Chimeric Mice with Thy1^−^ Mesenchymal Cells and Normal Lymphocytes Compared to WT Mice with Thy1^+^ Mesenchymal Cells and Thy1^+^ Lymphocytes

**Figure 3:**
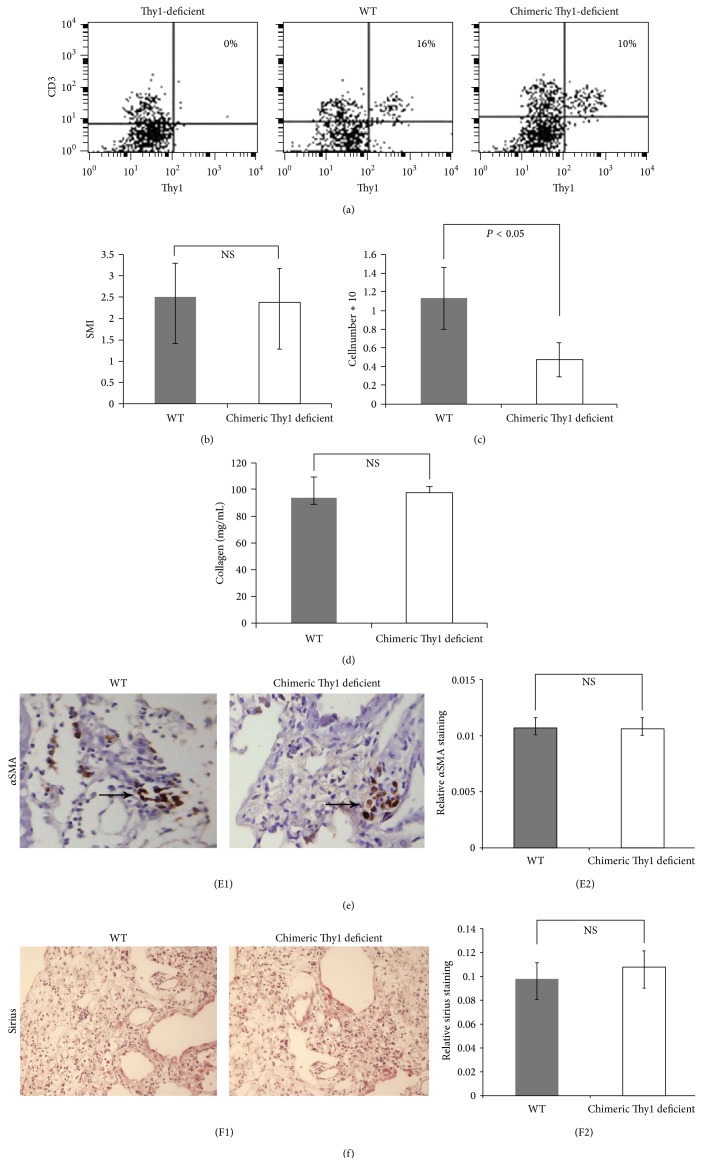
Assessment of fibrotic lung injury following bleomycin intratracheal instillation (IT) in wild-type mice with Thy1^+^ lymphocytes and Thy1^+^ myofibroblasts (WT), and chimeric Thy1-deficient mice with Thy1^+^ WT lymphocytes and Thy1^−^ myofibroblasts (chimeric). Thy1-deficient mice (DEF) were provided by Prof. R.J. Morris. Chimeric mice were created by whole-body irradiation (750 rad) of the Thy1-deficient mice, followed by transplantation with WT Thy1^+^ lymphoid cells (chimeric). Wild-type mice underwent whole-body irradiation and reconstitution with WT Thy1^+^ lymphoid cells as control (WT). (A) Flow cytometry analysis to detect Thy1^+^ immune cells in Thy1-deficient mice (DEF), wild-type mice (WT), and chimeric Thy1-deficient mice with transplanted WT Thy1^+^ lymphocytes. Thy1^+^ immune cells were identified in blood cells by double staining with PE-conjugated anti-Thy1 and FITC-conjugated anti-CD3. (B) Quantitative image analysis of pathological sections in chimeric Thy1-deficient mice (Thy1^+^ lymphocytes/Thy1– myofibroblasts, empty bars) and control WT mice (grey bars) 14 d following IT bleomycin. *n* = 6 WT and 8 chimeric 2 (C) Analysis of collagen content in lungs using the Sircol assay 14 d following IT bleomycin in chimeric Thy1-deficient (empty bars) and control WT mice (grey bars). (D) Total BAL cell count in lungs 14 d following IT bleomycin in chimeric (empty bars) and control WT mice (grey bars) (^*∗*^ *P* < 0.05) (E1, F1) *α*SMA and sirius staining in lung tissue sections 14 d following IT bleomycin of chimeric Thy1-deficient (empty bars) and control WT mice (grey bars). (E2, F2) 10 fields of every IHC-slide were digitized using the Ariol machine and semiquantitatively analyzed with the Ariol system. The relative *α*SMA and sirius staining ratios represent the ratio between numbers of stained and unstained cells in a certain analyzed area. Results were similar in 2 different experiments.

In order to specifically assess the contribution of myofibroblast Thy1 downregulation on lung inflammation and fibrosis in vivo in a system excluding Thy1 lymphocytes, we used chimeric Thy1-deficient mice. These mice exhibit completely Thy1^−^ mesenchymal cells (*α*SMA^+^ Thy1^−^), but normal, Thy1^+^ lymphoctyes (CD3^+^Thy1^+^) (Figure 3(a)). Assessment of lung fibrotic injury by semiquantitative morphological index (SMI) of pathological sections (Figure 3(b)), as well as collagen content (Figure 3(c)), showed no difference between WT and chimeric Thy1-deficient mice when compared to control saline-treated (not shown) mouse lungs; however, we detected a decrease in lung inflammation as determined by bronchoalveolar lavage (BAL) cellularity (Figure 3(d)). In addition, there were no differences between WT and Thy1-deficient mice in either the quantity of *α*SMA-positive cells examined by immunochemistry staining or in collagen I an IV staining, as examined by sirius red staining (Figure 3(E1), Figure 3(E2), Figure 3(F1), and Figure 3(F2)).

### 3.4. Increased CD4^+^/CD25^+^ T Regulatory Cells following IT Bleomycin in Chimeric Mice with Thy1^−^ Mesenchymal Cells and Normal Lymphocytes Compared to WT Mice with Thy1^+^ Mesenchymal Cells and Thy1^+^ Lymphocytes

**Figure 4:**
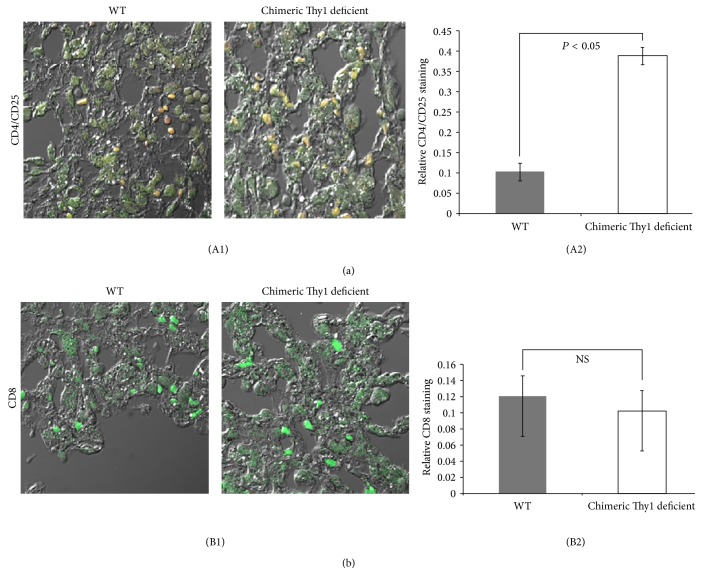
Assessment of the T cell population following bleomycin intratracheal instillation (IT) in wild-type mice (WT) (control) with Thy1^+^ lymphocytes and Thy1^+^ myofibroblasts, and chimeric Thy1-deficient mice with Thy1^+^ lymphocytes and Thy1^−^ myofibroblasts. Chimeric Thy1-deficient mice were created by total body irradiation (750 rad) of Thy1-deficient mice, followed by transplantation with WT Thy1^+^ lymphoid cells. For comparison, wild-type (WT) mice were irradiated and had adoptive transfer with Thy1^+^ (WT) lymphoid cells. (A1 and A2) Immunofluorescence of CD4^+^/CD25^+^, and (B1 and B2) CD8 staining of mouse lung sections 14 d following IT bleomycin in chimeric Thy1-deficient mice (empty bars) and control WT mice (grey bars). N 6 = 5 WT and 8 chimeric Thy1-deficient. (A1) Nomarski microscopy pictures of CD4/CD25 (yellow) (^*∗*^ *P* < 0.05), and (B1) CD8 (green) staining are presented. (A2 and B2) 10 fields of every immunofluorescence slide were digitized and semiquantitatively analyzed using the Ariol system. The relative staining ratio represents the ratio between numbers of stained and unstained cells in a certain analyzed are. The results were similar in 2 different experiments.

Having shown decreased inflammation in the lungs of chimeric Thy1-deficient mice compared to WT mice, we determined the differences in the inflammatory cell mileux of their lungs. We stained lung sections with anti-CD8 (cytotoxic cells) and costained them with anti-CD4 and anti-CD25 mAbs (T regulatory cells). A much larger number of CD4^+^/CD25^+^ lymphocytes were detected in chimeric Thy1-deficient mice when compared to WT mice (Figures 4(A1) and 4(A2)); however, there were a comparable number of CD8 cells in WT and Thy1-deficient mice (Figures 4(B1) and 4(B2)).

Taken together, these findings indicate that Thy1 on myofibroblasts in vivo has a role in the regulation of the inflammatory process, possibly by increased accumulation of T regulatory cells following bleomycin injury, with no effect on fibrosis development.

## 4. Discussion

The wide and diverse distribution of Thy1 suggests that it has distinct functions that vary between and in some cases even within cell types and tissues, and between similar tissues in different species, indicating that the biological role of Thy1 is context-dependent [31].

Using gene chip analysis, we show that Thy1 on lung myofibroblasts affects pathways of the nervous system as well as those affecting the development of malignant diseases (Table 1), as previously shown [32–35]. The number of genes that were downregulated by Thy1 crosslinking in lung myofibroblasts was higher than the number that were upregulated (Figure 2), indicating that Thy1 on lung myofibroblasts appears to be a suppressor factor rather than an activator in most cases. These observations are consistent with previous reports showing an inhibitory effect of Thy1 on cell outgrowth of neurites [33] and on tumor growth [32, 34, 35]. Gene expression studies following Thy1 crosslinking in vitro may indicate that Thy1 could bind to a fixed ligand in vivo, such as an integrin, and/or to another cell type, or perhaps to a matrix ligand. However, our understanding of Thy1 ligand and its functions is incomplete and warrants further study.

Our detection of the downregulation of myofibroblast Thy1 expression as fibrosis evolves is consistent with findings in several other studies of an inverse correlation between Thy1 expression on fibroblasts and the evolution of lung fibrosis [36, 37]. Of note, one laboratory reported that, under certain conditions, Thy1^+^ fibroblasts can produce more collagen compared to Thy1^−^ fibroblasts [38]. Our results also show that myofibroblast Thy1 crosslinking upregulates genes promoting inflammation and immunological functions (Table 3). In addition, Thy1 upregulated specific genes, including those of chemokines that are responsible for antigen-processing and presentation, as well as those of cytotoxic molecules that promote inflammation and immunological functions (Tables 2 and 3). In particular, we have shown here in the GeneChip analysis (Table 3), previously validated by us in Wb, FACS, and qPCR [28], that Thy1 activation upregulates FasL expression in lung myofibroblasts, which in turn promotes pro-inflammatory activity by the recruitment and activation of immune cells [39]. Through this upregulation, Thy1 also indirectly induces a cytotoxic cell phenotype in myofibroblasts. We have previously demonstrated that myofibroblasts from bleomycin-treated mice overexpress the FasL molecule and act as effector cells that induce apoptosis in Fas^+^ epithelial cells [24] and lymphocytes [21] via Fas/FasL interaction.

The stronger inflammatory response detected by BAL cellularity in control chimeric WT mice compared to chimeric mice with Thy1^−^ myofibroblasts and WT (Thy^+^) lymphocytes could be explained by GeneChip results, which show that Thy1 activation, as is the case in chimeric-WT mice, upregulates cytokine gene expression (Table 3). By upregulating cytokine gene expression, Thy1 may indirectly promote or induce myofibroblast recruitment of immune cells to the lungs. A lack of Thy1 on myofibroblasts, as is the case in Thy1^−^ chimeric mice, may reduce the level of chemokine protein expression in the lung following bleomycin IT, yielding lower BAL cellularity.

Thy1 molecule inhibited gene/s that can be profibrotic (Table 3). Its activation downregulated genes promoting myofibroblast cell proliferation, differentiation, and ECM component production. In addition, we showed that the fraction of the Thy1^+^ myofibroblast subpopulation is decreased at day 14 following bleomycin IT. This is consistent with previous reports showing that fibroblasts in the lungs of humans with IPF and of bleomycin-treated mice are predominantly Thy1^−^ [36]. Thus, absence of Thy1 in myofibroblasts may facilitate their faster proliferation, accumulation, and collagen production and therefore increase lung fibrosis.

The GeneChip analysis may provide some explanation for our in vivo results with chimeric Thy1-deficient mice, which showed no change in fibrosis but did show decreased inflammation, in comparison with chimeric control WT mice that had undergone similar adoptive transfer WT lymphocytes into irradiated WT host mice. These results indicate that when Thy1 is deficient in myofibroblasts, as is the case in chimeric Thy1-deficient mice, fibrotic features persist in an in vivo milieu with decreased inflammation.

Hagood et al. reported that Thy1-null mice develop more extensive and more severe lung fibrosis following bleomycin administration than do WT mice [36]. Although this is consistent with our in vitro results, which showed that Thy1 activation in lung myofibroblasts downregulates genes promoting cell differentiation and ECM production (Table 3), it is not consistent with our in vivo results showing no change in the fibrotic feature between chimeric WT- and Thy1-deficient mice. Thy1 is absent from all cells that would otherwise express it in Thy1-null mice, in contrast to our chimeric Thy1-deficient mice, which retain Thy1 expression on lymphocytes. Because Thy1 is normally expressed on murine lymphocytes, it is possible that the severe lung fibrosis following bleomycin IT that develops in Thy1-null mice is not due to changes in fibroblast Thy1 expression but rather is due to changes in lymphocyte function [40]. The role of lymphocytes in bleomycin-induced lung fibrosis remains controversial [41–45], as does the role of inflammation in IPF [19, 20, 41, 46–49]. Decreased inflammation following bleomycin injury, as is the case in the chimeric Thy1-deficient mice with Thy1-deficient myofibroblasts but Thy1^+^(WT) T cells, may suggest that myofibroblasts downregulate Thy1 expression as an additional mechanism allowing their accumulation, promoting immune modulation, and supporting disruption of immune surveillance [21]. Consistent with our results, which show similar fibrosis and similar augmentation of CD8 T cells in chimeric Thy1-deficient and control-WT mice (Figures 4(B1) and 4(B2)), it was shown that CD8^+^ T cells are associated with development and prognosis of fibrosis [50]. In addition, it was shown that a fibrotic environment in the lung results in an increased abundance of CD4^+^/CD25^+^ regulatory T cells [51]. Our findings show an increase in the number of CD4^+^/CD25^+^ regulatory T cells (Figures 4(A1) and 4(A2)) in chimeric Thy1-deficient mice, despite the fact that there was no difference in fibrosis between chimeric Thy1-deficient and WT mice. However, the two groups of mice differed in the extent of lung inflammation. It may be possible that differences in the accumulation of regulatory T cells in these mice cannot influence fibrosis, which was at its peak. However, the increase in T regulatory cells can, and did, have an impact on the extent of inflammation. This can be explained by the complicated interactions between regulatory cells, cytotoxic T cells, and myofibroblasts [52]. The role of Thy1 on lymphocytes remains a very interesting issue for further investigation. It may be possible to create chimeric mice with Thy-deficient lymphocytes and WT fibroblasts to pursue this research.

## 5. Conclusions

These findings indicate that, in lung fibrosis, lymphocytes increase Thy1 expression while the subset of Thy1^+^ lung myofibroblasts is decreased. Lung myofibroblasts downregulate Thy1 expression to increase their proliferative functions and to reduce the inflammatory milieu, possibly via induction of CD4^+^/CD25^+^ regulatory T cell accumulation, in vivo. Differences in the inflammatory responses of chimeric Thy1-deficient mice with Thy1^−^ myofibroblasts but Thy1^+^ lymphocytes versus WT mice do not affect fibrotic responses other than inflammation. These results indicate a newly identified role for Thy1^−^ myofibroblasts and Thy1^+^ lymphocytes.

## Figures and Tables

**Table 1:** Molecular pathways downregulated by Thy1.

Pathway name	# input genes in the pathway	*P* value
Long adhesion	13	1.02*E* − 06
Long-term depression	6	3.85*E* − 04
ECM-receptor interaction	6	6.59*E* − 04
Small cell lung cancer	5	0.004769
Regulation of actin cytoskeleton	8	0.005003
Prostate cancer	5	0.005006
Tight junction	6	0.007175
Melanoma	4	0.012134
Bladder cancer	3	0.015551
MAPK signaling pathway	8	0.016136
Axon guidance	5	0.022646
Endometrial cancer	3	0.027335
mTOR signaling pathway	3	0.027335
Gap junction	4	0.02851
Insulin signaling pathway	5	0.029243
Non-small cell lung cancer	3	0.030129
GnRH signaling pathway	4	0.031564
Alzheimer's disease	2	0.033214
Wnt signaling pathway	5	0.03603
p53 signaling pathway	3	0.048047
Glioma	3	0.048047
Cell cycle	4	0.048272
B cell receptor signaling pathway	3	0.049885
Long-term potentiation	2	0.049885
Neurodegenerative diseases	2	0.050764
Wnt signaling pathway	7	0.001962
B cell receptor signaling pathway	4	0.00836
VEGF signaling pathway	4	0.011283
Natural killer cell mediated cytotoxicity	5	0.01976
Hedgehog signaling pathway	3	0.026203
Neuroactive ligand-receptor interaction	7	0.036606
T cell receptor signaling pathway	4	0.037492
Dorsoventral axis formation	2	0.039028

**Table 2:** Molecular pathways upregulated by Thy1.

Pathway name	# input genes in the pathway	*P* value
Hematopoietic cell lineage	3	0.020743
ECM-receptor interaction	3	0.020743
Cytokine-cytokine receptor interaction	5	0.027124
Antigen processing and presentation	3	0.031685
Maturity onset diabetes of the young	2	0.003156
Autoimmune thyroid disease	2	0.026802

**Table 3:** Pathways involved in myofibroblast proliferation, differentiation, and interaction with the ECM and immune system that are affected by Thy1.

Category	Pathway	Gene name	Fold change^*∗∗∗*^
Proliferation	*Cell cycle *	Anaphase promoting complex subunit 2	−1.19
		Cyclin D2	−1.02
		CDC23 (cell division cycle 23 yeast homology)	−1.13
		Transformed mouse 3T3 cell double minute 2	−1.22
	*MAPK signaling pathway *	RIKEN cDNA 1500003O03 gene	−1.03
		Thymoma viral proto-oncogene 2	−1.12
		Activating transcription factor 4	−1.1
		Filamin C, gamma (actin binding protein 280)	−1.05
		Protein phosphatase 1B, magnesium dependent, *β* isoform	−1.06
		v-raf-leukemia viral oncogene 1	−1.07
		Serine/theonine kinase 3 (Ste20, yeast homolog)	−1.2
		Fibroblast growth factor receptor 1	−1.13
	*Insulin signaling pathway *	RIKEN cDNA 4932417H02 gene	−1.22
		Thymoma viral proto-oncogene 2	−1.12
		Brain glycogen phosphorylase	−1.37
		v-raf-leukemia viral oncogene 1	−1.07
		Sorbin and SH3 domain containing 1	−1.46
	*TGFβ signaling pathway *	Activin receptor IIA	−1.16
		Protein phosphatase 2	−1.19
		Thrombospondin 1	−1
	*Receptors *	Angiotensin receptor like 1	−1.85

ECM production and interaction	*Focal adhesion *	Thymoma viral proto-oncogene 2	−1.12
		Caveolin, caveolae protein 1	−1.13
		Cyclin D2	−1.02
		Collagen, type I, alpha 1	−1.02
		Collagen, type IV, alpha 1	−1.12
		Filamin C, gamma (actin bineing protein 280)	−1.05
		Integrin beta 1 (fibronectin receptor beta)	−1.04
		Laminin, alpha 2	−1.22
		v-raf-leukemia viral oncogene 1	−1.07
		Thrombonspondin 1	−1.19
		Talin 1	−1.17
		vav 1 oncogene	−1.59
		Vinculin	−1.3
	*ECM receptor interaction *	Collagen, type I, alpha 1	−1.02
		Collagen, type VI, alpha 1	−1.12
		Dystroglycan 1	−1.33
		Integrin beta 1 (fibronectin receptor beta)	−1.04
		Laminn, alpha 2	−1.22

Myofibroblast differentiation	*Regulation of actin cytoskeleton *	Actin, alpha 2, smooth muscle, aorta	−1.54
		Cofilin 1, non-muscle	−1.35
		Fibroblast growth factor receptor 1	−1.13
		Guanine nucleotide binding protein, alpha 13	−1.2
		Integrin beta 1 (fibronectin receptor beta)	−1.04
		v-raf-leukemia viral oncogene 1	−1.07
		vav 1 oncogene	−1.59
		Vinculin	−1.3
	*Differentiation *	MyoD1	−1.83
		Pax 7	−1.89

Inflammation and immunological function	*Hematopoietic cell lineage *	CD4 antigen	1.280914
		CD8 antigen, alpha chain	1.74495
	*Antigen processing* and *presentation *	Interferon alpha 13	1.57917
	*Cytokine-cytokine receptor interaction *	Small chemokine (C-C motif) ligand 11	1.913514
		Chemokine (C-C motif) ligand 17	1.571759
		Chemokine (C-X3-C motif) ligand 1	1.0517
		Interferon alpha 13	1.57917
		Nerve GFR (TNFR superfamily member 16)	1.451481
		Nos2	2.31
		Interleukin-1 receptor	−1
		FasL	1.71
		Interleukin-17 receptor	−1.2
		Cytokine receptor like	−1.17

^*∗∗∗*^Fold change is shown on a log_2_ scale.

## Abbreviations

BAL: Bronchoalveolar lavage
ECM: Extracellular matrix
FACS: Fluorescence-activated cell sorting
GPI: Glycosylphosphatidylinositol
IP: Intraperitoneal
IPF: Idiopathic pulmonary fibrosis
IT: Intratracheal instillation
PE: Phycoerythrin
qPCR: Quantitative polymerase chain reaction
RT: Room temperature
Thy1^−^: Thy1-negative
Thy1^+^: Thy1-positive
WT: Wild-type.
